# The sign of leser-trélat: think in the adenocarcinoma of the lung

**DOI:** 10.11604/pamj.2018.30.270.16337

**Published:** 2018-08-10

**Authors:** Hanane Asri, Mouna Soualhi

**Affiliations:** 1 Pulmonology Department, Mohammed V Military University Hospital, Faculty of Medicine and Pharmacy, Mohammed V University, Rabat, Morocco; 2 Pulmonology Department, Moulay Youssef Hospital, Faculty of Medicine and Pharmacy, Mohammed V University, Rabat, Morocco

**Keywords:** Leser trelat sign, lung cancer, seborrheic keratosis

## Image in medicine

The Leser-Trelat sign is the sudden appearance and rapid increase in the size and number of seborrheic keratoses in association with an underlying malignant disease. It is usually associated with adenocarcinoma, most frequently of the colon, breast, or stomach, exceptionally of the lung. We reported a case of patient 70-year-old man; one year ago, an increasing number of pruriginous skin lesions suddenly appeared. The progression was marked 4 months later by deterioration of the general state and dyspnea of exercise. Dermatologic examination revealed multiple seborrheic keratoses, a chest radiograph depicting complete right lung ate revealed a white lung, chest CT scan showing right lung atelectasis with obstruction of right main bronchus and right pleural effusion. The histopathologic study of the biopsy bronchial fibroscopy reported a lung adenocarcinoma. Patient was dead one week later. Our observation report association of the sign of leser-trélat with occult adenocarcinoma of the lung. Eruption of seborrheic keratoses has also been observed with benign neoplasms, pregnancy, human immunodeficiency virus infections, which indicate that the leser-trélat sign is not very specific. Despite these concerns, the eruption of multiple seborrheic keratoses should continue to trigger the thought of an internal malignancy in the differential diagnosis.

**Figure 1:**
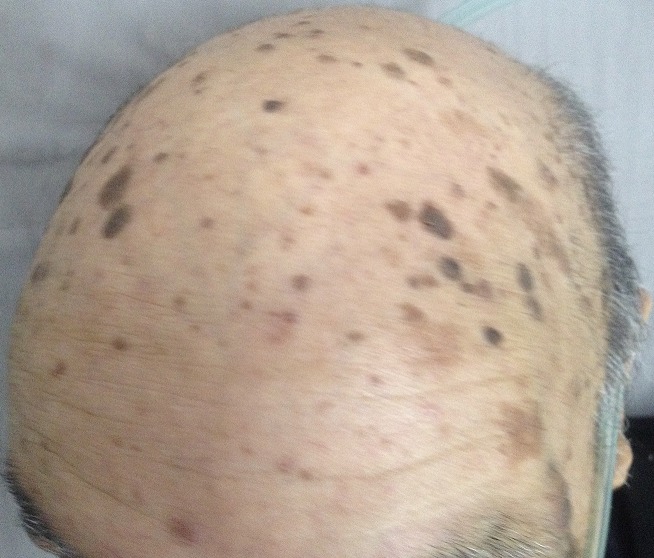
multiple seborrheic keratosis in forehead

